# Regarding: Regional variation in low-value musculoskeletal surgery: a nationwide study from the Finnish Care Register

**DOI:** 10.2340/17453674.2024.42635

**Published:** 2024-12-23

**Authors:** Ville PONKILAINEN, Anniina LAUREMA, Ville M MATTILA, Teemu KARJALAINEN

**Affiliations:** 1 Department of Orthopaedics and Traumatology, Tampere University Hospital; 2 Department of Surgery, Mikkeli Central Hospital, Mikkeli; 3 Department of Orthopaedics and Traumatology, Tampere University Hospital, Faculty of Medicine and Life Sciences, University of Tampere, Tampere, and COXA Hospital for Joint Replacement, Tampere; 4 Department of Surgery, Central Finland Central Hospital, Jyväskylä, Finland

*Sir*,—We extend our sincere thanks to Helka Koivu and coworkers for thoughtful engagement with our work and sharing their perspectives on the important topic of regional variation in low-value musculoskeletal procedures [1]. Such discussions are important for understanding research findings.

We would like to clarify a fundamental misinterpretation of our study. Our paper does not assert that ankle arthroscopy is categorically unnecessary but rather highlights the significant regional variations in its utilization. Such variation is a hallmark of inconsistent indications, often reflecting a lack of high-quality evidence driving clinical decisions. When procedures are unequivocally effective, variation tends to diminish, as seen in universally accepted treatments like insulin for type 1 diabetes. In contrast, the observed 10-fold differences in the incidence of assessed procedures strongly suggest overuse in certain areas, necessitating careful scrutiny of their indications and supporting evidence.

The authors of the letter allege 2 main methodological errors in our article:

1. Misinterpreting the evidence-based indications for ankle arthroscopy outlined by Glazebrook et al., thereby misclassifying ankle arthroscopy as low-value care, undermining its use while it is highly valuable to patients [2].
2. Using the Finnish Care Register for Health Care and claiming that the data is not reliable.

We disagree with both of these claims.

1. The authors cite that “Three indications for ankle arthroscopy received a grade B recommendation, meaning they were supported by good evidence from Level II or III studies.” However, what does “good evidence” from Level II or III studies mean when we are considering whether ankle arthroscopy is effective?

We question whether all authors of the letter have critically appraised the studies cited as evidence for the benefits of ankle arthroscopy. The referenced literature consists mainly of surgeon surveys, observational studies, and meta-analyses of such studies. These sources fail to demonstrate patient-important benefits when compared with open surgery, nor do they provide evidence favoring arthroscopy over no surgery at all. Given that other arthroscopic debridement procedures have turned out to be low-value care, the value of ankle arthroscopy for patients remains uncertain and merits further investigation. Moreover, merely showing non-inferiority to open surgery does not inherently signify high value to patients.

Perhaps the most striking claim in the letter is the assertion that “methods used in drug research are unsuitable for surgical outcomes.” We wonder whether the authors would make this case directly to the many scientists who have conducted high-quality surgical trials with placebo or nonoperative controls [3]. Establishing a procedure’s efficacy requires comparing it with placebo or usual nonoperative care—not assertions from a panel of keen arthroscopists. Observational studies and expert opinions, though informative, fall short of providing the rigorous evidence needed to validate surgery as effective.

As for the argument that the benefits of ankle arthroscopy are “too obvious” to justify RCTs, history cautions us against such assumptions. Similar beliefs have perpetuated the use of unvalidated practices in medicine, often to the detriment of patients. For conditions treated with ankle arthroscopy, rigorous experimental evidence is imperative to avoid repeating these mistakes and to ensure practices align with patient-centered outcomes.

2. The reliability of the Finnish Care Register for Health Care has been shown to be high in multiple studies [4-6] and no studies question the validity of the register or its use in this type of study setting. The authors of the letter claim that “many have found procedure codes false,” but it would be interesting to know who these “many” were, how many they were, and how they found the codes to be false? This is important, as hundreds of articles are based on this register.

Finally, we acknowledge that ankle arthroscopy may indeed provide benefits in certain scenarios and be useful in establishing exact diagnosis. However, determining its precise indications and effects requires well-designed trials. Until such evidence emerges, the broad implementation of this procedure is unlikely to improve population health. Should robust evidence become available, we would gladly support its use. Even then, however, our conclusions would remain valid: during 2020–2021, wide variation in the assessed procedures persisted, with small hospitals that had historically performed low-value surgeries continuing to do so at higher rates.
